# Comparison of classical multi-locus sequence typing software for next-generation sequencing data

**DOI:** 10.1099/mgen.0.000124

**Published:** 2017-07-04

**Authors:** Andrew J. Page, Nabil-Fareed Alikhan, Heather A. Carleton, Torsten Seemann, Jacqueline A. Keane, Lee S. Katz

**Affiliations:** ^1^​ Pathogen Genomics, Wellcome Trust Sanger Institute, Wellcome Genome Campus, Hinxton, Cambridgeshire CB10 1SA, UK; ^2^​ Microbiology and Infection, University of Warwick, Coventry, UK; ^3^​ Enteric Diseases Laboratory Branch, Centers for Disease Control and Prevention, Atlanta, GA, USA; ^4^​ Doherty Applied Microbial Genomics, Department of Microbiology and Immunology, University of Melbourne, Peter Doherty Institute for Infection and Immunity, Melbourne, Australia; ^5^​ Pathogen Informatics, Wellcome Trust Sanger Institute, Wellcome Genome Campus, Hinxton, Cambridgeshire, UK; ^6^​ Center for Food Safety, College of Agricultural and Environmental Sciences, University of Georgia, Griffin, GA, USA

**Keywords:** MLST, multi-locus sequence typing, software comparison, next-generation sequencing

## Abstract

Multi-locus sequence typing (MLST) is a widely used method for categorizing bacteria. Increasingly, MLST is being performed using next-generation sequencing (NGS) data by reference laboratories and for clinical diagnostics. Many software applications have been developed to calculate sequence types from NGS data; however, there has been no comprehensive review to date on these methods. We have compared eight of these applications against real and simulated data, and present results on: (1) the accuracy of each method against traditional typing methods, (2) the performance on real outbreak datasets, (3) the impact of contamination and varying depth of coverage, and (4) the computational resource requirements.

## Abbreviations

MLST, multi-locus sequence typing; NGS, next-generation sequencing; ST, sequence type; WGS, whole-genome sequencing.

## Data Summary

1. Simulated reads for datasets testing coverage and mixed samples have been deposited in Figshare; DOI: https://doi.org/10.6084/m9.figshare.4602301.v1.

2. Outbreak databases are available from GitHub; url – https://github.com/WGS-standards-and-analysis/datasets.

3. Docker containers used to run each of the applications are available from GitHub; url – https://github.com/andrewjpage/docker_mlst.

4. Accession numbers for the data used in this paper are available in the Supplementary Material.

## Impact Statement

Sequence typing is rapidly transitioning from traditional sequencing methods to using whole-genome sequencing. A number of *in silico* prediction methods have been developed on an *ad hoc* basis and aim to replicate classical multi-locus sequence typing (MLST). This is believed to be the first study to comprehensively evaluate multiple MLST software applications on real validated datasets and on common simulated difficult cases. It will give researchers a clearer understanding of the accuracy, limitations and computational performance of the methods they use, and will assist future researchers to choose the most appropriate method for their experimental goals.

## Introduction

A small number of bacterial foodborne pathogens, such as *Salmonella*, *Campylobacter*, *Listeria* and *Escherichia*, cause a huge burden of disease in humans and animals. With *Listeria monocytogenes*, although the case count is small, the case-fatality rate is high at approximately 21 to 38 % [1, 2] and a high economic burden [3]. In the US, each foodborne illness can cost anywhere from hundreds to millions of US dollars depending on the organism. Therefore, investigating potential foodborne outbreaks and preventing any illness is advantageous from both economic and public health standpoints. In order to understand these bacteria in more depth, there have been many studies to describe their population structure using phylogenetic methods based on multi-locus sequence typing (MLST) [4, 5].

Additionally, there have been many large-scale surveillance efforts for these pathogens. One of the most successful programs has been PulseNet International [6], which aids in the detection of common source outbreaks. Recently, large numbers of isolates have been subjected to whole-genome sequencing (WGS) through an initiative between the Centers for Disease Control and Prevention (CDC), the US Food and Drug Administration (FDA), the US Department of Agriculture (USDA), and the National Center for Biotechnology Information (NCBI). Through this collaboration, every *L. monocytogenes* genome that is discovered in the food supply, or in clinical samples, is being sequenced and uploaded to the NCBI Sequence Read Archive (SRA) database. This collaboration has since started sequencing a large percentage of *Escherichia coli, Salmonella enterica, Campylobacter coli, Campylobacter jejuni* and many others, with the eventual goal of completely switching from pulsed-field gel electrophoresis to WGS. In Europe, Public Health England sequences every *Salmonella* and *Mycobacterium tuberculosis* isolate submitted to them and deposits the data in the SRA. Perceiving a future need for worldwide collaboration on these new methods, the Global Microbial Identifier (GMI) [7] partnership was initiated in 2011 to encourage data sharing among all nations for many purposes, including public health and research.

To aid in population structure studies and in epidemiological investigations, MLST has been used for nearly two decades [8] to categorize different clonal expansions of these pathogens into broad categories, based on allelic variation amongst seven highly conserved housekeeping genes. Sequence typing can be performed using both next-generation sequencing (NGS) and classical sequencing techniques. Whilst MLST is a low-resolution classification compared to what is possible from NGS data, the nomenclature is in common usage by microbiologists and clinicians. A number of software applications have been developed using a variety of fundamentally different techniques to calculate sequence types (STs) from NGS data. However, there has been no comprehensive review to date on the accuracy, computational performance, robustness and ease of use of these methods. In this paper, we have evaluated multiple MLST software applications on a variety of datasets, both real and simulated, such as: (1) standard sets of outbreak data from the Gen-FS WGS Standards and Analysis Working Group (available from https://github.com/WGS-standards-and-analysis/datasets) [9], which includes *C. jejuni, E. coli, L. monocytogenes* and *S. enterica*; (2) *Salmonella* isolates that have been typed using both traditional capillary sequencing and NGS; (3) simulated reads of varying coverage; and (4) simulated mixed strains. Here, we describe a comprehensive list of command-line tools for MLST analysis and benchmark them with these standardized datasets in terms of accuracy and computer resources required.

## Software overview

MLST software can be categorized according to the input data they accept; there are tools that use raw sequence reads and tools that use *de novo* assemblies. Calling MLST from raw reads avoids the need to fully reconstruct the whole genome, theoretically allowing for a lower running time. However, in practice *de novo* assembly is routinely performed for bacteria [10] and assemblies may already be available for any given MLST analysis leading to faster sequence typing. The process of *de novo* assembly can introduce artefacts, particularly from short reads. For example, a gene may be fragmented over multiple contigs. A full overview is given in Table 1. In general, the desired characteristics of MLST software include:

**Table 1.** Overview of MLST software

**Software**	**Input**	**Algorithm**	**Licence**	**Source**	**Tests**	**Installation**	**Interface**
ariba	Reads	Assembly	GPL3	GitHub	Yes	Pip, Apt, Docker	Command line
BigsDB [11]	Contigs	blastn	GPL3	GitHub	No	Manual	Website
BioNumerics	Reads/ contigs	Proprietary/blastn	Bespoke	Proprietary	na	Manual	GUI
EnteroBase	Reads	ublast/usearch	na	na	na	na	Website
most [14]	Reads	Mapping	FreeBSD	GitHub	No	Manual	Command line
mlst*	Contigs	blastn	GPL2	GitHub	No	Brew	Command line
mlst-cge [16]	Contigs	blastn	Apache 2	Bitbucket	No	Docker	Command line/Website
MLSTcheck [17]	Contigs	blastn	GPL3	GitHub	Yes	CPAN, Docker	Command line
SeqSphere+ [18]	Contigs	na	Bespoke	Proprietary	na	Manual	GUI
SRST2 (24)	Reads	Mapping	BSD	GitHub	Yes	Apt, pip	Command line
stringMLST [21]	Reads	*k*-mer	Bespoke	GitHub	No	Manual	Command line

*https://github.com/tseemann/mlst

(1) high specificity of calling STs,

(2) resilience in the face of mixed samples,

(3) tolerance with low sequencing coverage,

(4) efficient usage of computational and disk resources,

(5) simple dependency management and installation,

(6) validated with automated tests to verify functionality works as intended,

(7) transparency of algorithm,

(8) and scalability to large numbers of isolates.

The interfaces to the software applications fall into two categories, those that operate on the command line and those that have a graphical interface. Command line input allows for high throughput analysis, but has a high barrier to entry for non-technical users. Graphical interfaces, such as websites, provide point and click interfaces that non-technical users find easier to use initially; however, they are often limited to the analysis of a few samples at a time. To reduce the impact of this limitation, some websites precompute results by downloading raw data directly from the short-read archives (EnteroBase: http://enterobase.warwick.ac.uk) [11].

Most of the software packages are available under open-source licences, with source code available in public repositories, such as GitHub (https://github.com). Source-code availability facilitates transparency for the underlying methods. Comprehensive automated tests, if designed correctly, ensure stability within software applications. Applications packaged for easy installation and dependency management such as: Apt (Debian), Homebrew, Docker, PyPy and cpan allow for the software to be installed in one step, allowing for immediate use by a range of users. An overview of MLST software applications follows.

Antibiotic Resistance Identification By Assembly (ariba) (https://github.com/sanger-pathogens/ariba) takes raw reads as input on the command line, and uses a combination of mapping and local *de novo* assembly to calculate alleles. Like srst2, it can be used more generally for gene detection and classification, allowing for antibiotic-resistance prediction, virulence-gene detection and plasmid replication gene classification. It is open source, has extensive unit tests and is packaged for easy installation.

Bacterial Isolate Genome Sequence Database (BigsDB) [11] is a web service whose primarily purpose is the management of sequence typing databases, as opposed to querying them. It is used by the majority of schemes as the backend for storing their typing data. The database can be queried in two ways, via a web interface or programmatically through a REST API. There is no described command line interface for queries; however, the mechanisms are in place to allow for it in the future. BigsDB can be used to create new MLST schemes.

BioNumerics (http://www.applied-maths.com/bionumerics) from Applied Maths is a commercial application that is widely used by public-health laboratories to calculate STs. Due to its proprietary nature, a full review is not possible; however, the authors described a reads-based *k*-mer sequence typing method in a patent [12] and do assembly-based sequence typing using blastn.

EnteroBase (http://enterobase.warwick.ac.uk) is a web resource that incorporates sequencing data from both public databases and directly from users for four genera (*Salmonella*, *Escherichia*, *Yersinia* and *Moraxella*), and assembles it *de novo* with an adjusted pipeline using SPAdes [13]. EnteroBase succeeds the University College Cork/Warwick MLST database (http://www.mlst.net/databases/), and maintains the database and assigns new alleles of MLST schemes for these genera. These data are mirrored through PubMLST via the EnteroBase API, which is available for all EnteroBase users. Alleles are called using nucleotide and amino acid sequence with usearch/ublast, which allows for high sensitivity for divergent allele variants. However, the source code is not publicly available.

Metric-Oriented Sequence Typer (most) [14] builds upon srst (version 1) [15] and uses a mapping-based approach to align alleles to reads, with a traffic light system indicating the confidence in the ST calling. One major difference to srst2 is that it takes a 100 base flanking region around the locus from a reference genome, reducing the impact of coverage drop off at the ends of the sequences. Additionally, it can assign predicted serovars to *Salmonella* isolates. It is used by Public Health England on clinical isolates and has strict, well-defined conservative criteria for calling STs to ensure accuracy. *mlst* (https://github.com/tseemann/mlst) takes *de novo* assemblies as input on the command line and uses blastn to align sequences to alleles. It is very fast and searches all databases on pubMLST to automatically detect the organism, then calculates the ST. Installation is very easy using *brew*.

*MLST* from the Center for Genomic Epidemiology (mlst-cge) [16] is a web-based method for calculating MLST. It can take assembled genomes or raw sequencing reads. If raw sequencing reads are provided, it performs a *de novo* assembly. Alleles are called using a blast-based method.

MLSTcheck [17] takes *de novo* assemblies as input on the command line and uses blastn to align sequences to alleles. It is packaged for easy dependency installation, and has unit test coverage. It produces a multi-fasta alignment of concatenated allele sequences for each sample, which allows for phylogenetic trees to be easily reconstructed. Novel allele sequences are saved to allow for them to be submitted to the MLST curators.

SeqSphere+ [18] from Ridom is a commercial application that is widely used by public-health laboratories. It uses assembled sequences to call STs. It is packaged for easy installation and consists of a large suite of analysis pipelines for automated sequence analysis. Due to its proprietary nature, a full review is not possible.

Short Read Sequence Typing 2 (srst2) [19] takes raw reads as input on the command line and uses a mapping-based approach to align reads to the alleles. It is packaged for easy dependency installation and is widely used for a variety of applications in addition to MLST including: antibiotic-resistance prediction, virulence-gene detection and serotyping [20]. The software licence is free for both commercial and non-commercial use, and it has unit tests.

stringMLST [21] takes raw reads as input on the command line and uses *k*-mers to detect MLST alleles. Instead of detecting allele coverage or parsing for potential SNPs, an allele call is made by identifying the allele with the most number of matching *k*-mers. The use of *k*-mers gives a substantial speed advantage, but at the expense of accuracy. This method is fast enough to detect STs in real time during sequencing, so it holds much promise for the future. It is free for non-commercial purposes and it has no automated tests.

The described applications were optimized to work with MLST. Their performance on higher resolution schemes, such as ribosomal MLST, core genome MLST, and whole genome MLST, is quite different, with most scaling poorly to schemes with hundreds or thousands of genes, as this was a case the applications were never fundamentally designed to handle. Alternative methods are required to cater for these cases; thus, extended schemes are not covered in this paper.

## Database availability

The availability of databases containing alleles and ST profiles for different species is an important aspect of any MLST software application as outlined in Table 2, since this dictates how easy it is to use the software. These databases also need to be kept up to date, as the underlying schemes are constantly being extended as new isolates are sequenced. Out of date databases can mean that rapidly emerging clonal expansions may be missed, impairing epidemiological investigations. ariba, BioNumerics, *mlst*, MLSTcheck, stringMLST, SeqSphere+ and srst2 all provide automated scripts/methods to download all of the latest databases from pubMLST [11], which are immediately ready to use. This provides immediate access to schemes for over 125 species. *mlst* and stringMLST go one step further and additionally bundle all available databases in their software repository, which are regularly updated. most does not provide an automated method for downloading new or updated databases, instead directing researchers to a set of manual steps. They do provide a small number of bundled databases (six and nine, respectively); however, these only represent a fraction of the currently available databases on pubMLST. The databases bundled with most were last updated in December 2015, so are missing all recent updates and additions to the schemes, including new STs, so researchers cannot be certain novel results are indeed novel.

**Table 2.** Overview of the MLST databases available with each software application.

**Software**	**Automated download**	**Bundled DBs**	Age of bundled DBs*	**DBs ready to use**
ariba	Yes	0	–	Yes
BioNumerics	Yes	0	–	Yes
*mlst*	Yes	125	1 month	Yes
MLSTcheck	Yes	0	–	Yes
most	No	6	>1 year	Yes
SeqSphere+	Yes	0	–	Yes
srst2	Yes	0	–	Yes
stringMLST	Yes	128	1 month	Yes

DB, Database.

*The age of the bundled databases was calculated on the 15 March 2017.

## Evaluation

A full comparison could only be performed with the six open-source command line MLST software applications, ariba (v2.7.2), *mlst* (v2.8), MLSTcheck (v2.1.1630910), most (v 2e3da07), srst2 (v0.2.0) and stringMLS*T* (v0.3.6). Comparisons of the accuracy of results were performed for the two commonly used commercial applications, BioNumerics (v7.6.2) and Ridom SeqSphere +v4.0.0 (2017–04); comparable computational performance evaluations were not possible; however, these are secondary to accuracy. BigsDB and EnteroBase were excluded as they are web services with extensively featured pipelines and the computational performance of the MLST calling component could not be measured independently. mlst-cge was excluded because an essential internally hosted software repository was unavailable at the time of testing. Partial results are available for EnteroBase for some datasets, where relevant.

Each application was evaluated on four different datasets, two real and two simulated. Dataset 1 contained 85 samples from standard sets of outbreak data from the Gen-FS WGS Standards and Analysis working group (available from https://github.com/WGS-standards-and-analysis/datasets). Dataset 2 consisted of 72 *Salmonella* samples from EnteroBase, which represent samples that have both MLST data based using traditional capillary sequencing and using Illumina NGS technologies. Dataset 3 consisted of artificially generated reads with varying levels of coverage. From this, the minimum sequence depth required for each software application could be calculated. Dataset 4 consisted of artificially generated reads from two different *Salmonella* serovars where all alleles differ, mixed in different ratios out of a total depth of coverage of 50×. The accuracy of applications could then be determined with mixed samples (a common case) and the point at which the mixed samples became detectable.

The experiments for Dataset 1 were performed using the CDC compute infrastructure. For the rest of the experiments [2–4], we used the MRC climb OpenStack cloud [22] as the base platform for the evaluations. Each of the applications was run in their own Docker container [23] available from GitHub (https://github.com/andrewjpage/docker_mlst). The Debian Testing distribution was used as the base operating system for all containers as it provides access to a large range of up-to-date bioinformatics software. The host VM had four cores and 32 GB of RAM running Ubuntu 16.04 (LTS); however, only a single core was used for the evaluations. All datasets used for this analysis are available for download as described in the data bibliography or from the public archives using the accession numbers in the Supplementary Material. Where assemblies were required as input to MLST applications, the raw reads were *de novo* assembled with SPAdes (v3.9.0) [13] using the default parameters. SPAdes was chosen as it is widely used and consistently produces high-quality results on bacterial data [24]. All experiments using the two commercial applications, BioNumerics and SeqSphere+, were performed using the CDC compute infrastructure with default options and the SPAdes assemblies as described above.

## Real outbreak datasets

Standard datasets (https://github.com/WGS-standards-and-analysis/datasets), covering *L. monocytogenes* from stone fruit [25]*, E. coli* from sprouts [26], *C. jejuni* from raw milk (http://www.outbreakdatabase.com/details/hendricks-farm-and-dairy-raw-milk-2008/) and *S. enterica* from spicy tuna [27], comprising 85 samples, were analysed by each of the software applications. These are real outbreak datasets where there were substantive epidemiological investigations and full details are available [9]. No false positives were reported by any application, they made either the correct call, a low-confidence call or no call. A summary of the overall performance is provided in Table 3, with extended details available in Table S1 (available with the online Supplementary Material). There was a wide variation in the results, with only three applications (stringMLST, BioNumerics and MLSTcheck) correctly calling all of the STs. most failed to confidently call any of the spicy tuna *Salmonella* samples, but did identify the correct STs, flagged as low confidence (amber). There was a 29-fold variation in the running times between the applications (stringMLST vs srst2) using raw reads as input (Table 1). This extra computation imposes financial costs and increases the analysis time after sequencing.

**Table 3.** Summary of performance of each algorithm on real outbreak data for four different species (85 samples)

Software	Total time (min)	Correct ST (%)	No call/low confidence (%)
ariba	109.5	98.8	1.2
BioNumerics	na	**100**	**0**
mlst*	1.9 (+2873)	96.5	3.5
most†	1189.7	49.4	50.6
MLSTcheck*	63.8 (+2873)	**100**	**0**
SeqSphere+	na	96.5	3.5
srst2	2380.2	95.3	4.7
stringMLST	**80.8**	**100**	**0**

Values in bold indicate the best results in each column.

*The time to assemble with SPAdes before running the applications was 2873 min and is included separately.

†most identified the correct ST in 97.6 % of cases, but flagged 48.2 % of these calls as low confidence.

## Comparison to capillary data

This dataset consisted of 72 *Salmonella* samples that had been sequenced using traditional capillary sequencing (originally deposited in http://mlst.warwick.ac.uk, now available through EnteroBase) and sequenced using NGS. This allowed for technology independent validation of the NGS MLST software applications. The samples covered a wide range of *Salmonella*, from hosts including humans, reptiles, birds and farm/domestic animals, and from the environment, collected between 1940 and 2014. The dataset contained an estimated 32 different STs, with 38 of the samples predicted to have a serovar of Typhimurium, which causes severe disease in a wide range of hosts, including humans. Full details of the samples (including accession numbers) and results are in Table S2. The ST calls matched in 89 % (64/72) of cases between the capillary data and the NGS MLST software applications, which additionally includes MLST results from the EnteroBase website. Two samples (RKS1252 and RKS1256) were suspected sample swaps with each other. The sample E698 differed between the capillary sequencing results and all other methods with no overlapping alleles. It is possibly a sample swap with another unknown sample or the original sample contained multiple strains. For OLC-1602 and 556-59/192, six out of seven alleles matched in all of the results, but the capillary sequencing data reported a single different allele. Whilst capillary sequencing data is recognized by the community as a gold standard, it is not error free [28], with calls sometimes made using a single read, leaving little resilience to sequencing errors. As the NGS data had very high depth of coverage (over 30×) of this allele, it is likely that the NGS results were correct. Nearly all of the calls from most were low confidence (rated amber); however, they correlated with the results from the other applications, and it is just that most has very stringent, validated, criteria for calling an ST. Three samples were flagged by multiple applications as problematic; however, in every case the capillary sequencing data, stringMLST, EnteroBase, SeqSphere+ and BioNumerics confidently called an ST, indicating a contaminant has been missed. Eight applications flagged sample 139K as problematic; however, stringMLST confidently called an ST, indicating overconfidence in ST calling. MLSTcheck and BioNumerics called a different ST for 2 samples; however, this appears to be due to duplicate allele profiles in the underlying database at pubMLST. Overall, we conclude that whilst the MLST results between capillary sequencing data and NGS data are nearly identical, the MLST based on NGS data is more accurate and reliable when presented with edge cases.

## Impact of depth of coverage

**Fig. 1.**
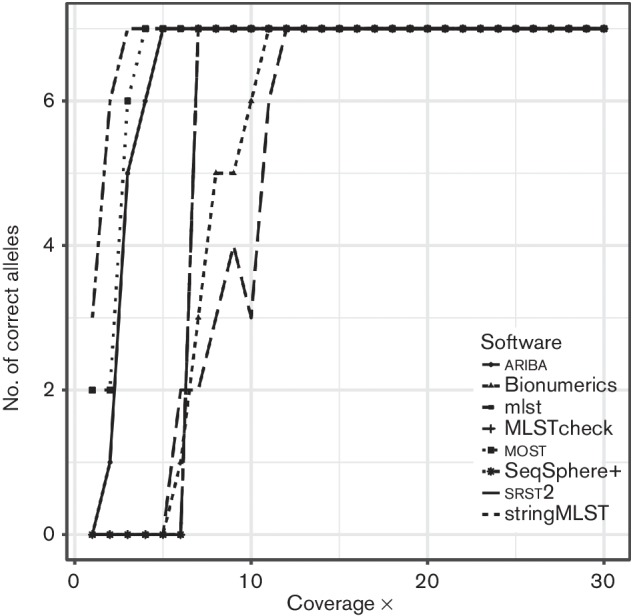
Number of correct calls of each application as coverage increases. Each ST consists of seven alleles, and all seven must be correctly and confidently called to calculate a ST.

The impact of depth of coverage over the MLST genes was assessed by artificially generating perfect paired-ended reads with a length of 125 bases and a median insert size of 400 bases with varying levels of coverage using fastaq (v3.14.0). The allele sequences plus 500 base flanking regions were extracted from *S.* *enterica* Typhi CT18 [29], accession number AL513382, and artificial paired-end reads were generated with mean depths of coverage from 1× to 30×. The simulated reads were free from sequencing errors to allow for the effect of coverage alone to be measured. Therefore, the minimum effective depth of coverage for each application could be tested. All applications could accurately call STs when the coverage was greater than 12×; however, below this the minimum depth of coverage applications required varied greatly, as shown in Fig. 1. stringMLST correctly called the ST with just 3× coverage; however, it gave false-positive results for lower coverage alleles. ariba correctly called the ST from 5× with no false-positive results. SRST2 correctly called the ST from 12× coverage with no false-positive results; however, it did correctly identify the ST from 6× with low confidence.

**Fig. 2.**
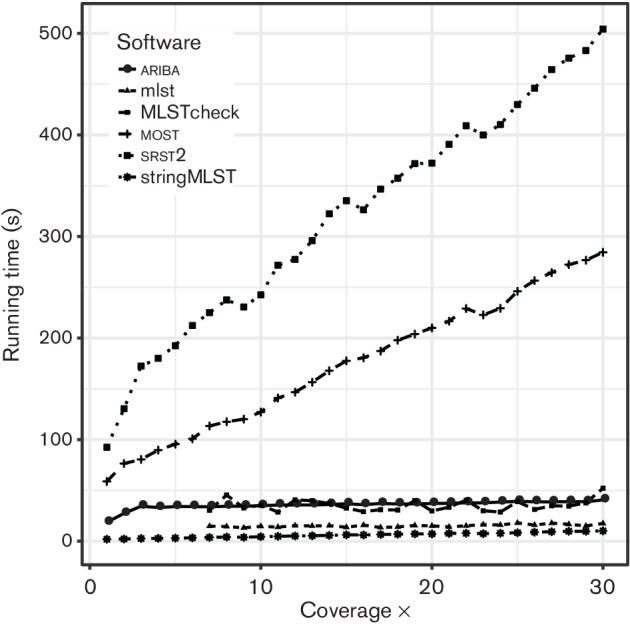
Running time (s) of each application as the coverage increases to assess the impact of the depth of coverage. No assembled contiguous sequences could be generated where the coverage was less than 7×, as such no data was recorded for the reliant methods (*mlst* and MLSTcheck). No performance results are available for BioNumerics or SeqSphere+.

**Fig. 3.**
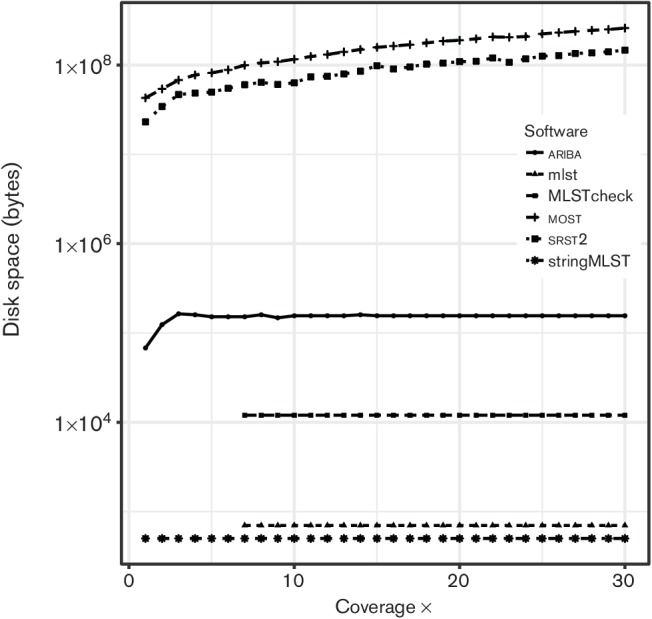
Disk space requirements in bytes for each software application as the depth of coverage increases. Due to the large difference between applications, a log scale is used.

The computational resources required varied greatly with stringMLST taking just 10 s to call an ST with 30× coverage, as shown in Fig. 2, and the final disk space requirements were negligible, as shown in Fig. 3. Whilst minimizing the disk space resources needed for the application is generally positive, stringMLS*T* does not output enough information about the allele calls to allow for further analysis, for example, to interrogate a false-positive result. The time to call an ST at 30× with ariba was 40 s with 0.1 Mbytes output data. The disk-space requirement is higher than stringMLST, but provides the allele assemblies used to call the ST, which is useful for further analysis. srst2 is an order of magnitude slower, taking over 500 s to call an ST at 30×. The disk space required for the final output is also very substantial at 147 Mbytes, which equates to a storage cost of 475 bytes per base of sequencing as shown in Fig. 3. While most confidently correctly called each individual allele from 4×, the overall ST call was flagged as low confidence below 10× due to its inherently conservative nature. The running time given for *mlst* and MLSTcheck includes the *de novo* assembly time with SPAdes, which accounts for most of the running time. MLSTcheck takes on average four times longer (25 s per sample) to return a result than *mlst* (5.9 s per sample), with the final results between the two being identical.

## Impact of mixed samples

Contamination and mixed colonies are a standard complexity in microbiology [30]. To understand the behaviour of the different MLST software applications in the presence of more than one strain, we constructed a simulated dataset consisting of two *Salmonella* samples with different alleles in varying ratios. This allowed us to see at what point contamination/mixed strains becomes detectable. Once detected, we would expect an MLST application to flag the results as low confidence or provide no result at all to avoid false positives. The flip side of this is that if algorithms are too sensitive to low level contamination and sequencing errors, they become less useful on real world applications, so need to be tolerant to some low-level noise.

**Fig. 4.**
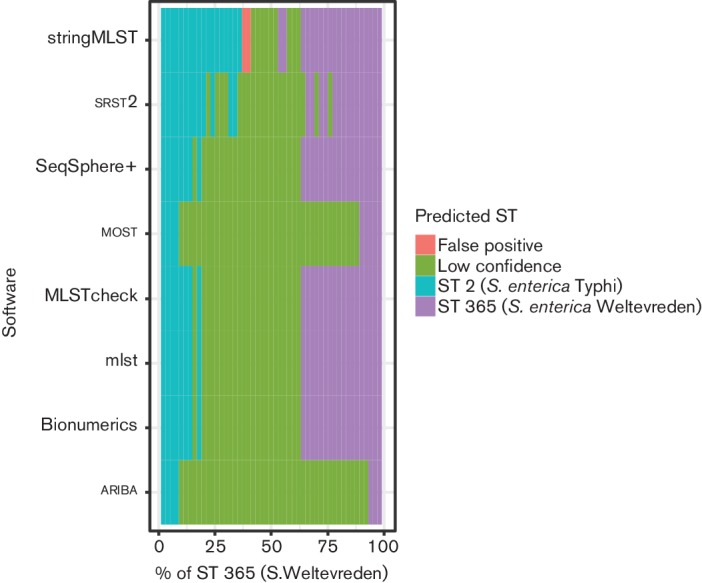
STs called by each software application when given data containing two different *Salmonella* samples in varying ratios of abundance. Where there is no ST called, or where the ST has any ambiguity at all, it is marked as low confidence. A false positive is where an ST is called with high confidence and is not one of the two samples in the raw data.

The allele sequences plus 500 base flanking regions were extracted from *S. enterica* Typhi CT18 [29], accession number AL513382, and *S.* *enterica* Weltevreden 10 259 [31], accession number LN890518. Artificial paired-end reads were generated using fastaq to give a total coverage of 50×, beginning with CT18 at 1× and 10 259 at 49× in a single fastq file. The coverage of each sample was varied in steps of 1× to generate a dataset of 49 fastq files. Fig. 4 shows that the accuracy of the software varies, but follows a general pattern, calling the sample with the highest coverage at the highest levels, with uncertainty in the middle as the proportion of the two samples becomes similar. The worst case is where a software application calls an ST with high confidence that is not in the underlying data (false positive), and only occured with stringMLST. most and ariba are highly conservative, detecting that there are mixed samples when the samples are at very low levels of coverage (at 4–5×). MLSTcheck, *mlst,* SeqSphere*+* and BioNumeric*s* all performed identically, with the performance linked to how well SPAdes assembled the underlying genomes. There was no clear boundary with srst2 and it varied between high-quality calls and low-confidence calls as the mixing of the samples changed.

## Conclusion

It is clear that not all MLST calling applications function as expected. Problems with some software include: out of date databases, computationally inefficient methods, false-positive results, inability to call alleles at low coverage and variable performance in the presence of mixed samples. Therefore, there is scope for improvement. Overall though, these software applications’ ST calls using NGS data are concordant with traditional MLST calling methods based on capillary sequencing data, perform moderately well with low mean genome coverage, and are sometimes able to report low confidence when faced with contamination.

## Data bibliography

1. Parkhill J. *Salmonella enterica* subsp. enterica serovar Typhi CT18, EMBL AL513382 (2002).
2. Andrew J. Page. *Salmonella enterica* subsp. enterica serovar Weltevreden 10259, EMBL LN890518 (2016).

## Supplementary Data

42 Supplementary File 1
